# Detection of Walk Tests in Free-Living Activities Using a Wrist-Worn Device

**DOI:** 10.3389/fphys.2021.706545

**Published:** 2021-08-12

**Authors:** Daivaras Sokas, Birutė Paliakaitė, Andrius Rapalis, Vaidotas Marozas, Raquel Bailón, Andrius Petrėnas

**Affiliations:** ^1^Biomedical Engineering Institute, Kaunas University of Technology, Kaunas, Lithuania; ^2^Faculty of Electrical and Electronics Engineering, Kaunas University of Technology, Kaunas, Lithuania; ^3^Biomedical Signal Interpretation & Computational Simulation (BSICoS) Group, Aragón Institute of Engineering Research (I3A), IIS Aragón, University of Zaragoza, Zaragoza, Spain; ^4^Centro de Investigación Biomédica en Red en Bioingeniería, Biomateriales y Nanomedicina (CIBER-BBN), Zaragoza, Spain

**Keywords:** 6-min walk test, functional capacity, functional performance, functional status, physical activity, fitness tracker, wearable device, fitbit

## Abstract

Exercise testing to assess the response to physical rehabilitation or lifestyle interventions is administered in clinics thus at best can be repeated only few times a year. This study explores a novel approach to collecting information on functional performance through walk tests, e.g., a 6-min walk test (6MWT), unintentionally performed in free-living activities. Walk tests are detected in step data provided by a wrist-worn device. Only those events of minute-to-minute variation in walking cadence, which is equal or lower than the empirically determined maximal SD (e.g., 5-steps), are considered as walk test candidates. Out of detected walk tests within the non-overlapping sliding time interval (e.g., 1-week), the one with the largest number of steps is chosen as the most representative. This approach is studied on a cohort of 99 subjects, assigned to the groups of patients with cardiovascular disease (CVD) and healthy subjects below and over 40-years-old, who were asked to wear the device while maintaining their usual physical activity regimen. The total wear time was 8,864 subject-days after excluding the intervals of occasionally discontinued monitoring. About 82% (23/28) of patients with CVD and 88% (21/24) of healthy subjects over 40-years-old had at least a single 6MWT over the 1st month of monitoring. About 52% of patients with CVD (12/23) and 91% (19/21) of healthy subjects over 40-years-old exceeded 500 m. Patients with CVD, on average, walked 46 m shorter 6MWT distance (*p* = 0.04) compared to healthy subjects. Unintentional walk testing is feasible and could be valuable for repeated assessment of functional performance outside the clinical setting.

## 1. Introduction

Exercise testing to assess the response to physical rehabilitation, medical or lifestyle interventions requires serial tests of functional capacity, and thus presents important constraints (Arena et al., 2007; Forman et al., 2017). To assess functional capacity, a ventilatory expired gas analysis should preferably be performed, requiring specialized equipment and advanced supervision. Given that physiological limits should be reached while working on an ergometer or a treadmill, the test cannot be properly completed by many older individuals due to fatigue or pain, also by those markedly deconditioned, e.g., suffering from heart failure or chronic obstructive pulmonary disease (Fletcher et al., 2013).

Well-established walk tests do not demand maximal physical efforts, and thus are convenient, safe, and inexpensive alternatives accessible to most except severely impaired individuals (Solway et al., 2001). In a fixed-time walk test, the longest distance an individual can walk under the encouragement of a supervising staff member is measured and compared with the individual-specific reference distance. Various time intervals have been considered over the years, namely, 2, 6, and 12 min, of which a 6-min walk test (6MWT) has been settled down as a reasonable compromise between too short and too exhausting (Heresi and Dweik, 2011). Since the guidelines on the 6MWT were published (American Thoracic Society, 2002), the test has been playing an important role in evaluating functional capacity, predicting outcomes, and assessing treatment efficiency across a variety of pulmonary and cardiac conditions (Enfield et al., 2010; Bellet et al., 2012; Yazdanyar et al., 2014; Bohannon and Crouch, 2017; Harmsen et al., 2017; Parry et al., 2019).

Given that walk tests reflect the integrated response of many systems involved during physical activity, including cardiovascular, respiratory, and muscle metabolism, relationships with various measures have been identified (Singh et al., 2014). For instance, the distance walked during the 6MWT shows moderate to strong correlation with maximal oxygen uptake (Carter et al., 2003; Turner et al., 2004; Ross et al., 2010), peak work (Carter et al., 2003; Turner et al., 2004) and physical activity measures (Mainguy et al., 2011; Hill et al., 2012). Meanwhile, slow cadence, which in turn results in shorter walked distances, is associated with midlife aging and lifelong brain health (Rasmussen et al., 2019), all-cause, cardiovascular and cancer mortality (Stamatakis et al., 2018), and is one of the primary indicators of frailty syndrome (Dent et al., 2019; Stenholm et al., 2019). Considering that the 6MWT is a submaximal exercise, it should not be viewed as an inferior alternative to the more demanding tests, but rather as a complementary approach to assessing the ability of an individual's to perform daily activities.

Walk tests are administered in clinics, hence, at best repeating every few months or once a year might not be enough aiming at early detection of functional loss. Thanks to the advent of wearable technology, it is now possible to perform walk tests outside the clinical environment and collect information on disease progression or training efficiency at more frequent time intervals. Several studies have employed wearable devices to estimate the distance walked during a walk test either by using integrated GPS tracking (Wevers et al., 2011; Salvi et al., 2020, 2021) or inertial sensors (Jehn et al., 2009, 2013; Schulte et al., 2012; Cheng et al., 2013; Brooks et al., 2015; Capela et al., 2015; Prescher et al., 2016; Ata et al., 2018; Burton et al., 2018; Blagev et al., 2019; Salvi et al., 2020, 2021). While the majority of research assessed wearable-based approaches by performing a supervised walk test in a lab, few attempts have been made to study self-administered testing, during which individuals actively decide to take a test at a place of their convenience (Brooks et al., 2015; Salvi et al., 2020, 2021). Encouragingly, the self-administered 6MWT is sufficiently accurate, reproducible, and acceptable by both healthy individuals and those with varying severity of congestive heart failure (Brooks et al., 2015); however, unintentional walk testing, accomplished by analyzing free-living physical activity data, has not received research attention yet, though has the potential to better reflect daily functional status.

Former research, often clinician-administered and performed in a lab or under controlled conditions, did not explore the feasibility to detect unintentional walk tests in free-living activities, where the episodes of physical activity are of different intensity and occur unpredictably. Accordingly, the main goal of the present study is to propose and examine a novel approach to walk testing which enables the estimation of distance walked during unintentionally performed walk tests. The study reflects on the findings in free-living step data acquired using a wrist-worn device without introducing any wearer intervention. To better understand walk tendencies, subjects of a wide age range, subdivided into the groups of healthy individuals and those with cardiovascular disease (CVD), were included in the study. The potential applications of the proposed approach include the following: (i) assessment of functional performance outside the clinical setting; (ii) remote monitoring of patients with cardiorespiratory disease aiming at early detection of functional loss; and (iii) conducting large-scale longitudinal studies for the purpose of exploring the relationships between walk properties and health risks.

## 2. Materials and Methods

### 2.1. Study Population and Data Acquisition

The study cohort consists of 99 subjects (63 women) assigned to the groups of healthy individuals and patients with CVD following standard clinical examination. Patients with CVD were diagnosed with chronic diseases, namely, ischemic heart disease, chronic heart failure, hypertensive heart disease, and persistent atrial fibrillation. Taking into account that the ability to walk fast depends on age and starts to decrease after the age of 40 (Lopopolo et al., 2006), healthy subjects were further subdivided into age groups of ≥ 40 and <40 years. Subject characteristics are given in Table 1.

**Table 1:** Subject characteristics in three groups.

	**Patients**	**Healthy**	**Healthy**
	**with CVD**	**(≥ 40 years)**	**(< 40 years)**
Number of subjects (women)	28 (16)	24 (14)	47 (33)
Age, years	56.0 (38–68)	49.5 (40–68)	30.9 (19–39)
Height, cm	173.5 (156–191)	170.0 (158–191)	172.0 (158–195)
Weight, kg	79.5 (54–132)	80.5 (54–111)	74.3 (52–130)
Body mass index, kg/m^2^	26.7 (19.1–37.3)	28.6 (20.7–34.4)	25.0 (17.4–34.9)

*Certain parameter values are given as median (range)*.

The data were originally collected by the information technology company dHealthIQ Care (Utrecht, the Netherlands) and the primary health care clinic Signata (Kaunas, Lithuania). The subjects were randomly selected and recruited voluntarily among the visitors of Signata from the fourth quarter of 2017 to the fourth quarter of 2019. The subjects were instructed to wear a commercial wrist-worn device Fitbit Charge 2 or Fitbit Alta HR (Fitbit, San Francisco, CA, the US) for at least 7 days, except when showering, bathing, or swimming, while maintaining their usual physical activity regimen. About 78 subjects wore Fitbit Charge 2 and 21 wore Fitbit Alta HR. Both wrist-worn devices provide minute-to-minute steps and heart rate at intervals of 5 s or longer, depending on the signal quality (Rapalis et al., 2018).

Signed written consent to participate in the study was obtained from all subjects. The study was conducted in accordance with the ethical principles of the Declaration of Helsinki (64th WMA General Assembly, Fortaleza, Brazil, October 2013). Identifiable information was removed from the collected data to ensure subject anonymity.

### 2.2. Detection of an Unintentional Walk Test

Given that 6 min is the most widely applied walk test duration, the methodology to detect unintentional walk tests is viewed from the perspective of the 6MWT; however, the methodology is not confined to this specific test duration, making 2 min (2MWT) and 12 min (12MWT) also suitable.

An unintentional 6MWT is detected in step data, provided by a wrist-worn device on a minute-to-minute basis, using a sliding 6 min window with 1 min overlap. To ensure that only fast walking is represented by the walk test, running, defined as >150 steps per min (Abel et al., 2011), and ordinary slow walking, defined as ≤ 60 steps per min, are assumed to represent unusual walking cadence for the walk test. In addition, the candidate walk tests which contain the device-detected stair climbing are also considered unsuitable, and thus excluded by the algorithm.

**Figure 1:**
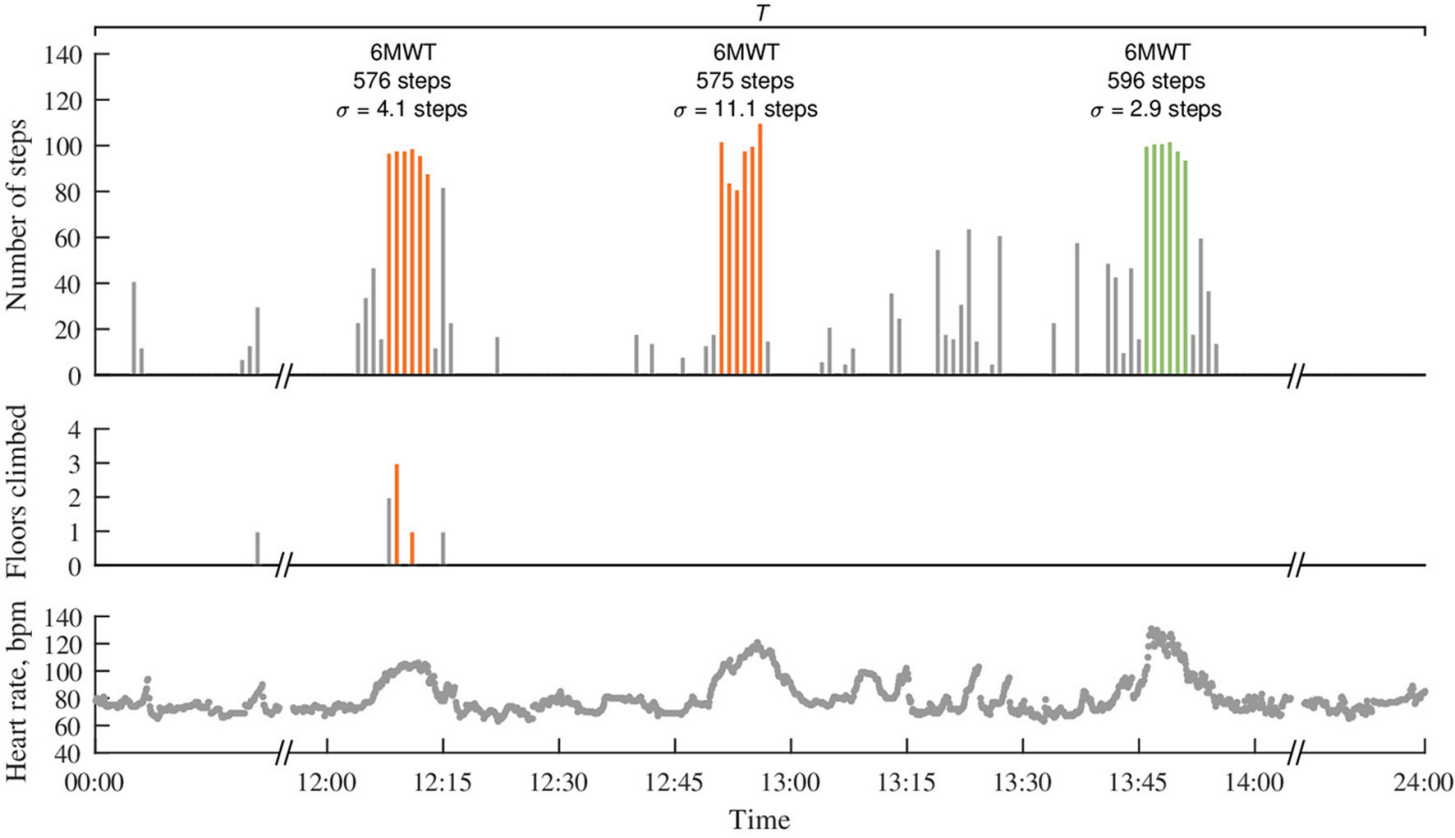
Detection of an unintentional 6MWT in minute-to-minute step data acquired in free-living activities using a wrist-worn device. The most representative 6MWT with the largest number of steps is depicted in green, whereas 6MWTs of other candidate, which either have a smaller number of steps or do not satisfy the determined criteria (e.g., contain stair climbing or cadence variation σ > 5 steps), are depicted in orange. Note that heart rate is not used for the detection of candidate walk tests but only serves for determining the subject-specific reference walking distance, to which the estimated distance is judged, refer to the Equation in (2).

Following the requirements for the 6MWT (American Thoracic Society, 2002), the test should be performed on a flat surface in a straight path, resulting in a steady cadence over the entire walk test (Motl et al., 2012; Dalgas et al., 2014). In free-living activities, cadence varies due to intervals of stops, decelerations, accelerations, and turns; therefore, it is reasonable to assume that a nearly consistent cadence indicates straight walking. Accordingly, only a walking interval with a minute-to-minute step variation, thereafter cadence variation σ, equal or lower than the empirically determined maximal SD, is considered as a walk test candidate. Ultimately, out of all detected walk test candidates over the non-overlapping sliding time interval *T* (e.g., 1 day, 3 days, and 1 week), the one with the largest number of steps is chosen as the most representative, refer to Figure 1. That is, either one or none representative walk test is possible at each time interval *T*.

### 2.3. Estimated Walking Distance

To compare with the reference walking distance, which is often given in meters, the number of steps in the 6MWT is converted to the distance by estimating a stride length for each subject. The conversion relies on the finding that cadence and stride length are linearly related to the cadence of up to 150 steps per min (Egerton et al., 2011). Based on the regression equations derived (Egerton et al., 2011), after modifications to include the height of the subject, the walking distance over 1 min is estimated as follows:

(1) $$\begin{array}{r} d=\{\begin{array}{c} \left(0.0031n^{2}+0.086n\right)h,\text{ for }a\;<\;65\;\text{years},\\ \left(0.0028n^{2}+0.098n\right)h,\text{ for }a\;\geq \;65\;\text{years}, \end{array} \end{array}$$

where *n* is the total number of steps per min, *h* is height of a subject in meters, and *a* – age in years.

The estimated walking distance, denoted by *6MWD*_*e*_, is found by adding up *d* estimated in each minute of the 6MWT.

### 2.4. Reference Walking Distance

The reference walking distance (*6MWD*_*r*_) against which the *6MWD*_*e*_ is judged depends on various variables, such as age, height, weight, and gender. While it is well-known that a stride length is often greater in taller individuals, explanation of other determinants is less evident. The influence of advancing age on a shorter walking distance is attributable to the reduction in muscle mass and strength (Song and Geyer, 2018), whereas being overweight probably has a negative effect due to lower lean muscle mass. Taking this into account, the multiple regression equation derived for healthy women and men of age 42–76-year-old is chosen as the reference walking distance in meters (Casanova et al., 2011):

(2) $$\begin{array}{r} 6MWD_{r}=361-4a+200h+300\frac{hr_{m}}{hr_{p}}-1.5w, \end{array}$$

where *hr*_*m*_ is maximal heart rate during the 6MWT, *hr*_*p*_ is age-depended maximal predicted heart rate, estimated as 210 − *a* × 0.79 for women and 221 − *a* × 0.95 for men (Sydó et al., 2014), and *w* is weight of a subject in kilograms. Since men usually walk larger distances than women (Chetta et al., 2006; Casanova et al., 2011), the *6MWD*_*r*_ is corrected by subtracting 30 m for women based on the findings in Casanova et al. (2011).

### 2.5. Statistical Analysis

In the first set of analyses, characteristics of walk tests unintentionally performed in free-living activities are explored. The number of detected walk tests is given for various combinations of *T* and σ. Also, the number of detected 6MWTs for different *T* is given for each study subject. Relationships between *6MWD*_*e*_ with and without σ restriction, as well as between actual wear time and *6MWD*_*e*_, and between the number of walk test candidates and *6MWD*_*e*_, are assessed using the Spearman's correlation coefficient.

In the second set of analyses, the within-subject reproducibility of *6MWD*_*e*_ during unintentional walk tests is assessed using the coefficient of variation *C*_υ_, defined as the ratio of the SD to the mean of the estimated walking distances. To increase the robustness of *C*_υ_ estimation, at least three detected walk tests per subject are required. The reproducibility results are summarized using boxplots.

In the third set of analyses, the groups of subjects are compared with respect to *6MWD*_*e*_. The Shapiro-Wilk test was used to assess data normality, and, because of normal distribution, *6MWD*_*e*_ is summarized using the mean and the 95% CI. Given that the difference between *6MWD*_*e*_ and *6MWD*_*r*_ can be positive as well as negative, these differences are represented with boxplots. The Student's *t*-test for independent samples was used to compute the *p*-value.

## 3. Results

### 3.1. Monitoring Characteristics in Free-Living Activities

Monitoring characteristics in free living activities for different subject groups are given in Table 2. The total monitoring time was 12,471 subject-days. Some subjects discontinued monitoring occasionally, resulting in 8,864 subject-days of actual wear time. The total monitoring duration exceeded 14 days in 86% (24/28) of patients with CVD and 94% (67/71) of healthy subjects. Meanwhile, the actual wear time exceeded 14 days only in 57% (16/28) of patients with CVD and 87% (62/71) of healthy subjects.

**Table 2:** Monitoring characteristics in three groups.

	**Patients with CVD**	**Healthy (≥ 40 years)**	**Healthy (<40 years)**
Monitoring duration, days	18.5 (6–753)	44.0 (14–599)	56 (6–808)
Actual wear time, days	13.7 (1–599)	21.9 (7–434)	39.8 (3–686)
Number of subjects who exceeded monitoring duration/actual wear time of			
7 days	26/22	24/24	46/44
14 days	24/16	24/21	43/41
1 month	9/8	14/11	37/32
3 months	8/7	3/2	21/16

*Certain parameter values are given as median (range)*.

### 3.2. Walk Tests in Free-Living Data

Table 3 summarizes the results of detected unintentional walk tests in free-living activities for different test durations. As expected, the number of detected walk tests highly depends on the chosen analysis time interval *T* and maximal cadence variation σ, being the largest for *T* = 1 day when no σ restriction is applied. In patients with CVD, *T* = 1 day with no σ restriction results in 7.6 6MWTs per subject-month on average; while, *T* = 1 week and σ ≤ 1 step reduce the number of eligible 6MWTs to only 0.8 per subject-month. The numbers given for the 2MWT and the 12MWT indicate that the 2MWT is 2–7 times more common than the 6MWT, whereas the 12MWT is 2–3 times less common depending on the chosen values of *T* and σ. Comparison of patients with CVD with healthy subjects shows that the number of detected 2MWTs are similar in both groups. On the other hand, patients with CVD have a considerably lower number of 6MWTs, which can be explained by their inferior functional status, making it difficult to walk for longer periods. No notable difference in the number of detected walk tests is observed between healthy subjects below and over 40 years.

**Table 3:** The average number of detected walk tests in free-living activities for different analysis time intervals *T* and cadence variations σ.

	**2MWT**				**6MWT**				**12MWT**			
	**No σ**	**σ ≤ 5**	**σ ≤ 3**	**σ ≤ 1**	**No σ**	**σ ≤ 5**	**σ ≤ 3**	**σ ≤ 1**	**No σ**	**σ ≤ 5**	**σ ≤ 3**	**σ ≤ 1**
*CVD*												
*T* = 1 day	18.7	16.6	15.3	11.7	7.6	3.8	3.0	1.7	2.7	1.6	1.3	0.8
*T* = 3 days	7.5	7.3	7.2	6.5	4.7	2.8	2.1	1.1	1.8	1.1	0.8	0.5
*T* = 1 week	3.4	3.3	3.3	3.3	2.8	1.9	1.5	0.8	1.3	0.7	0.6	0.4
*Healthy ≥40 yr*												
*T* = 1 day	17.6	15.8	14.7	12.6	9.9	6.5	5.2	3.3	5.6	2.8	2.2	1.0
*T* = 3 days	7.4	7.1	7.0	6.5	5.6	4.4	3.7	2.5	3.7	2.2	1.8	0.9
*T* = 1 week	3.4	3.3	3.3	3.2	2.9	2.8	2.5	1.8	2.3	1.6	1.4	0.7
*Healthy <40 yr*												
*T* = 1 day	17.7	15.5	14.5	12.3	9.3	6.9	5.7	3.9	4.8	3.1	2.5	1.3
*T* = 3 days	7.5	7.3	7.1	6.6	5.4	4.1	3.5	2.6	3.0	2.1	1.7	1.1
*T* = 1 week	3.5	3.5	3.4	3.4	3.0	2.5	2.2	1.7	1.9	1.4	1.2	0.8

*The results are obtained covering the entire monitoring duration*.

*In this table, “No σ” means that no restriction to maximal cadence variation is applied*.

**Figure 2:**
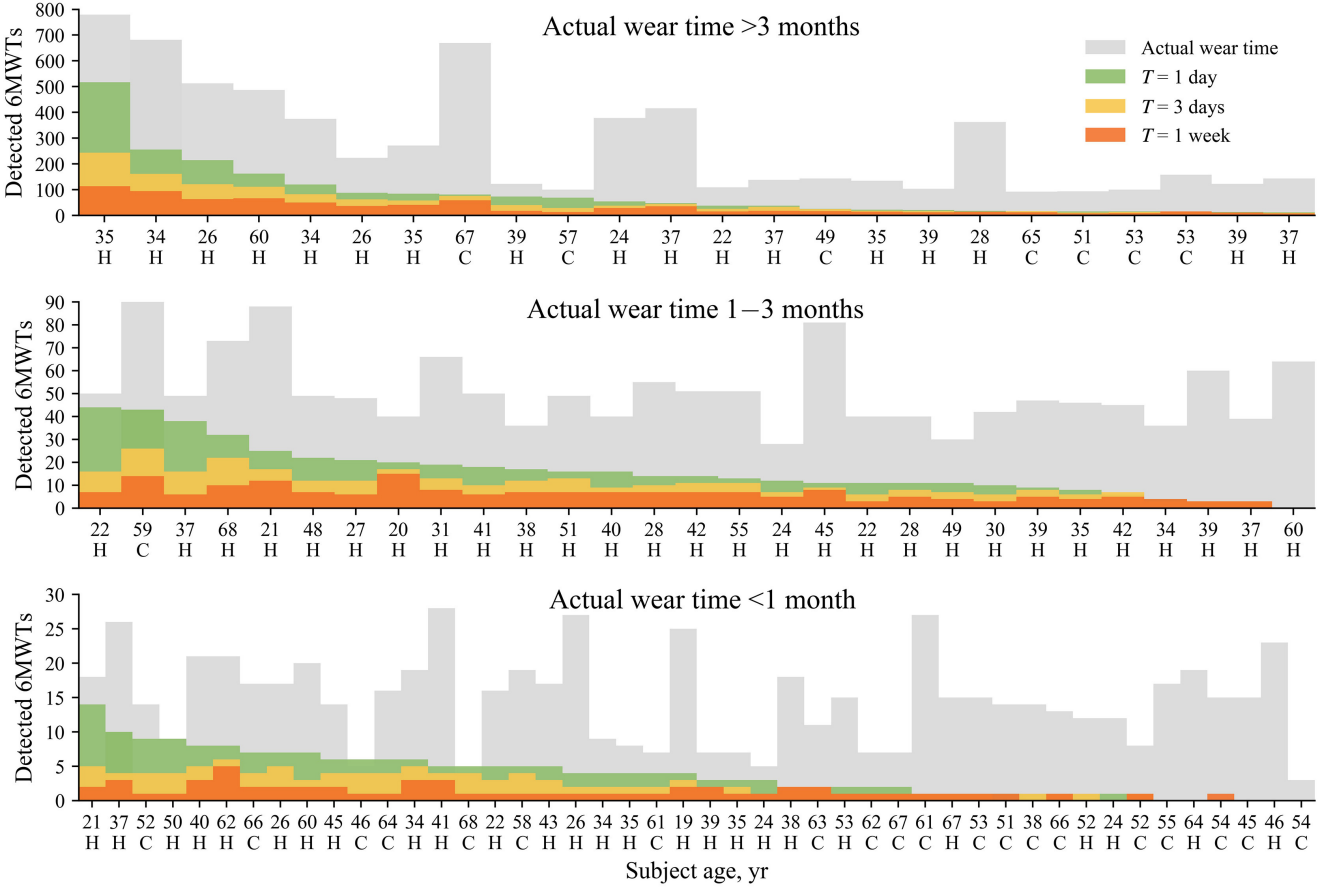
The number of detected 6MWTs for different analysis time intervals *T* for each study subject. Subjects are subdivided based on the actual wear time depicted in gray. Data are sorted with respect to the number of detected 6MWTs for *T* = 1 day. The cadence variation σ ≤ 5 steps. In this figure, “C” denotes a patient with a CVD, whereas ‘H’–denotes a healthy subject.

Figure 2 shows the number of detected unintentional 6MWTs for each study subject when different analysis time interval *T* is applied. To facilitate visual analysis, the subjects are further subdivided based on the actual wear time, i.e., >3 months, 1–3 months, and <1 month. Most subjects, i.e., 82% (23/28) of patients with CVD, 88% (21/24) of healthy subjects over 40 years, and 96% (45/47) of healthy subjects below 40 years had at least a single eligible 6MWT over the entire monitoring duration. The number of detected 6MWTs tend to increase for longer device wear duration, however, this tendency is only obvious when the actual wear time exceeds 3 months, whereas no such correlation is observed for < 1 month. The latter finding can be explained by the older and less physically active subjects who committed to at least 7-day monitoring but did not engage in physical activities intensive enough to be considered walk test candidates.

**Figure 3:**
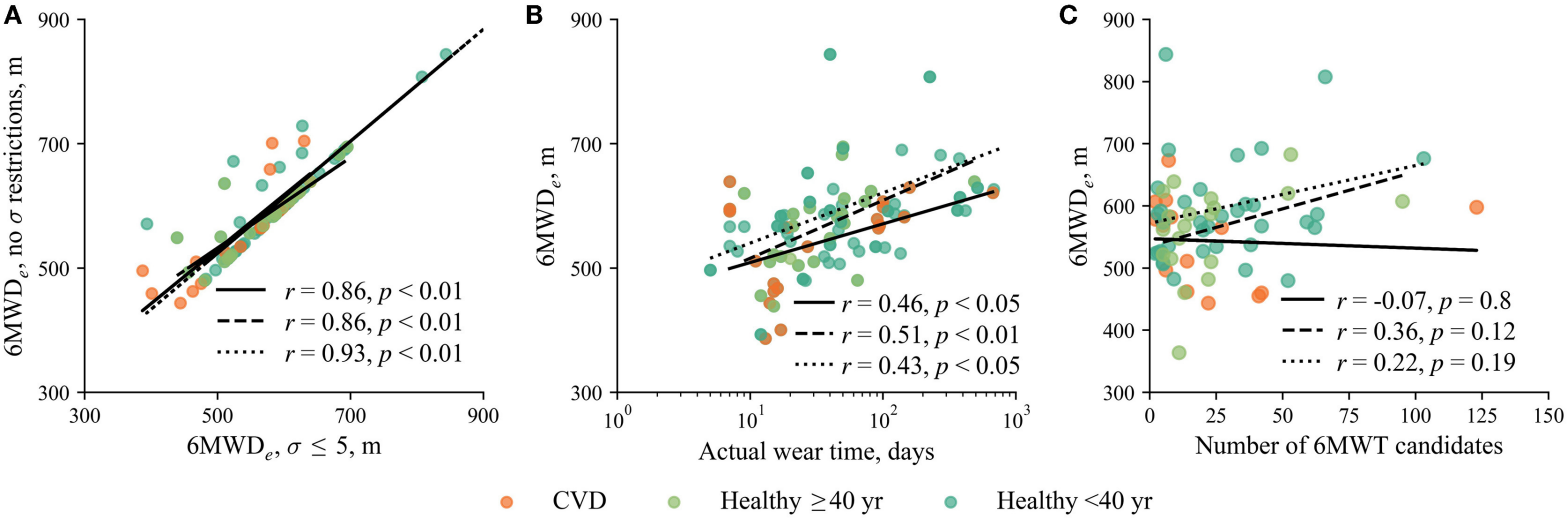
**(A)** The relationship between the *6MWD*_*e*_ with and without restriction to cadence variation. **(B)** The relationship between the *6MWD*_*e*_ and actual wear time when σ ≤ 5 steps. **(C)** The relationship between the *6MWD*_*e*_ and the number of 6MWT candidates when σ ≤ 5 steps. Solid, dashed, and dotted regression lines represent patients with CVD, healthy subjects over 40-years-old, and healthy subjects below 40-years-old, respectively. In this figure, analysis time interval *T* = 1 week, resulting in 23 patients with CVD, 21 healthy subjects over 40-years-old, and 45 healthy subjects below 40-years-old, who had at least a single eligible 6MWT. The 6MWT with the largest *6MWD*_*e*_ over the entire monitoring duration is selected. Note that the beginning and the end of the regression line are determined by the group of subjects it applies to.

Figure 3A shows the influence of the restriction to cadence variation on the estimated walking distance *6MWD*_*e*_ when comparing to the *6MWD*_*e*_ without any cadence restriction. The restriction of cadence variation to σ ≤ 5 steps only marginally affects the *6MWD*_*e*_ in most cases; however, the difference exceeds 50 m in 15% of subjects who had at least a single eligible 6MWT, suggesting that the cadence restriction may result in considerable underestimation of *6MWD*_*e*_ in some subjects. Figure 3B shows a tendency toward a larger *6MWD*_*e*_ for increasing actual wear time, which is expected, since longer wear time increases the likelihood of detecting the walking event satisfying the requirements for the unintentional 6MWT. Meanwhile, the number of 6MWT candidates has no significant influence on the *6MWD*_*e*_ (Figure 3C).

### 3.3. Reproducibility of the Estimated Walking Distance

**Figure 4:**
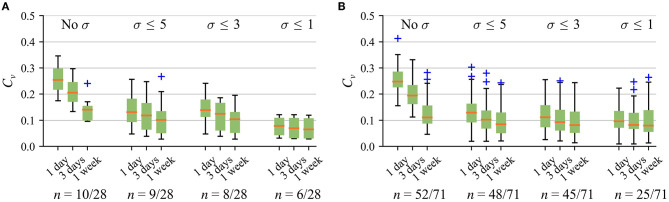
Within-subject reproducibility for the 6MWT with different cadence variation σ in **(A)** patients with CVD and **(B)** healthy subjects below and over 40-years-old. Only those subjects who meet the criteria of at least three walk tests detected are included for *C*_*v*_ estimation. In this figure, “No σ” means that no restriction to maximal cadence variation is applied.

To resemble a clinical walk test, and also increase the reproducibility of the *6MWD*_*e*_, it is desirable to ensure a relatively constant cadence over the test duration. As expected, the best within-subject reproducibility, indicated by the lowest coefficient of variation *C*_υ_, is obtained for the strictest maximal cadence variation σ ≤ 1, refer to Figure 4. However, low σ markedly reduces the number of detected walk tests since only few subjects meet the criteria of at least three walk tests required to compute *C*_*v*_. For instance, by choosing *T* = 1 week and σ ≤ 1 step, at least three 6MWTs were detected in only 21% (6/28) of patients with CVD and 35% (25/71) of healthy subjects. Reproducibility can be improved by using larger *T* since more walk test candidates are available to choose from.

**Figure 5:**
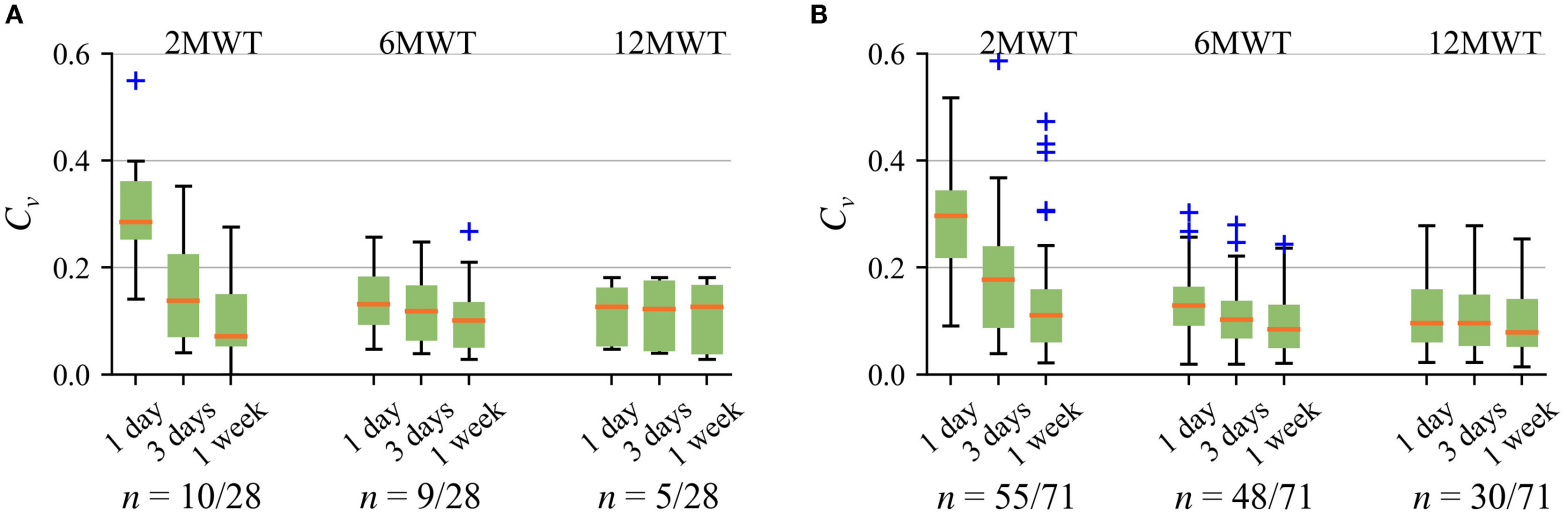
Within-subject reproducibility using different walk test duration for different analysis time interval *T* (σ ≤ 5 steps) in **(A)** patients with CVD and **(B)** healthy subjects below and over 40-years-old. Only those subjects who meet the criteria of at least three walk tests detected are included for *C*_υ_ estimation. In this figure, “No σ” means that no restriction to maximal cadence variation is applied.

Figure 5 indicates that the best overall reproducibility, including patients with CVD and healthy subjects, is obtained for the 6MWT when *T* = 1 week. Accordingly, based on the above-discussed aspects and the possibility to cover weekdays and weekends by the analysis time interval, the following parameter values were chosen as a trade-off: σ ≤ 5 steps and *T* = 1 week. These values are used in the rest of the article.

### 3.4. Comparison of Patients With CVD With Healthy Participants

**Figure 6:**
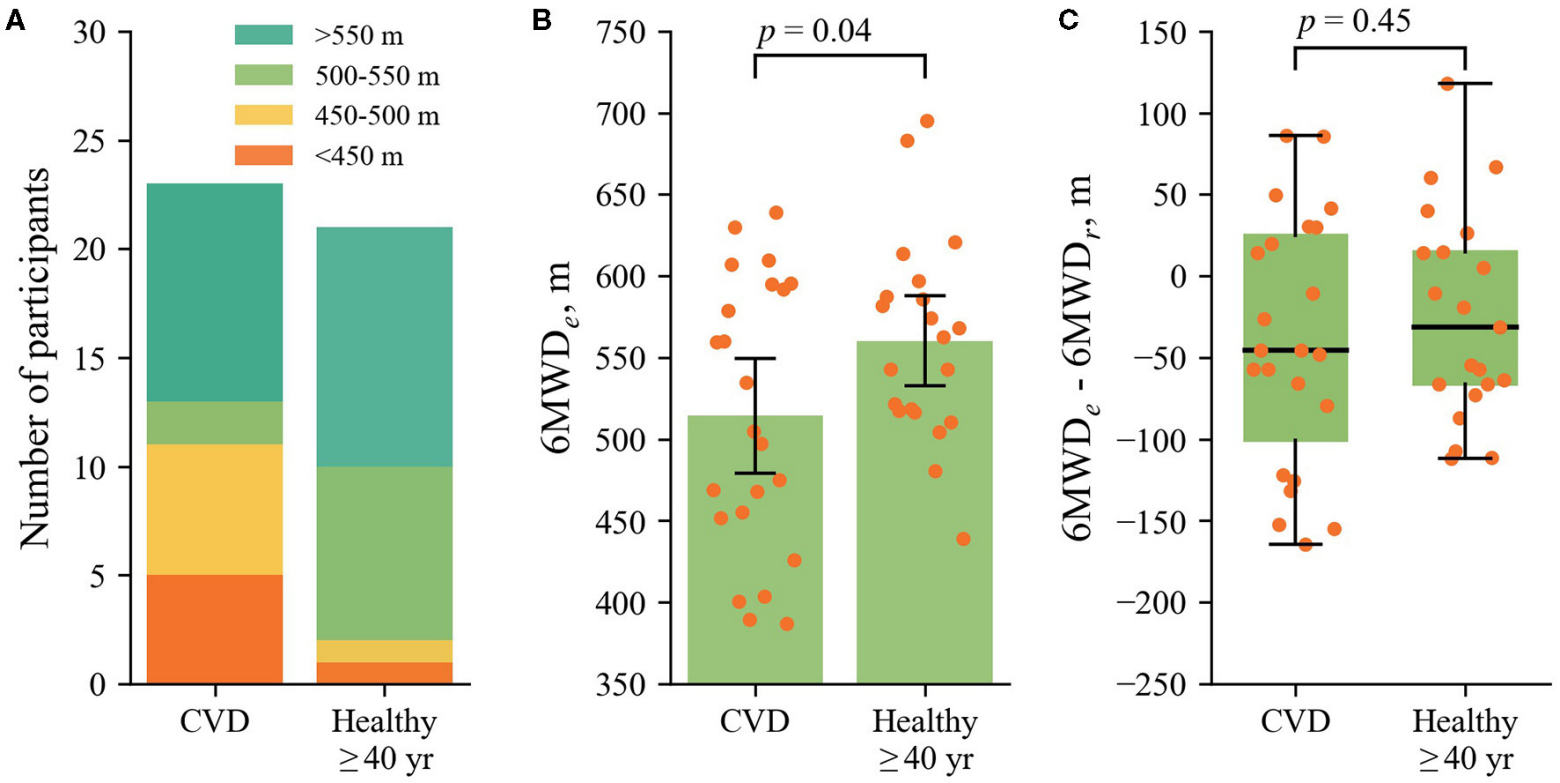
**(A)** Stacked diagrams of the *6MWD*_*e*_ for the different groups of subjects. **(B)** The *6MWD*_*e*_ for the different groups, where the dots display individual *6MWD*_*e*_ values. **(C)** The difference between the *6MWD*_*e*_ and the *6MWD*_*r*_. The group of healthy subjects below 40 years is not included since the equation for the reference walking distance is not validated for younger individuals.

Figure 6 compares patients with CVD and healthy subjects over 40 years with respect to the *6MWD*_*e*_ as well as the difference between the *6MWD*_*e*_ and the *6MWD*_*r*_. The results include only the first monitoring month to ensure similar conditions between the groups. 23/28 patients with CVD and 21/24 healthy subjects older than 40 years had at least a single 6MWT over the 1st month. Half of patients with CVD (12/23) succeeded to exceed 500 m, whereas this distance was exceeded by most of healthy subjects over 40 years (19/21) (Figure 6A). Patients with CVD, on average, walked 46 m shorter distance than healthy subjects older than 40 years (Figure 6B). The *6MWD*_*e*_ was lower than the *6MWD*_*r*_ for most of the subjects in both groups. The median deficiency was 45.3 m for patients with CVD and 31.3 m for healthy subjects over 40-years-old. Correlation between the *6MWD*_*e*_ and the *6MWD*_*r*_ was 0.39 (*p* < 0.01), 0.23 (*p* = 0.07), and 0.42 (*p* < 0.05) for patients with CVD, healthy subjects over 40 years, and healthy subjects below 40 years, respectively.

## 4. Discussion

To our knowledge, this is the first study that explores a completely unobtrusive approach to long-term monitoring of functional performance through walk tests unintentionally performed in free-living activities. The study provides insights into the minimal monitoring duration required to accomplish unintentional walk testing. On average, at least two 6MWTs per month are expected for the recommended parameter set (*T* = 1 week and σ ≤ 5 steps). Hence, 2 weeks of actual monitoring time are needed to detect a single unintentional walk test. Indeed, severely ill or frail individuals might find even 6 min of walking too exhausting and have less motivation to wear the device for extended periods. For such patients, 2MWT could be a more appropriate choice (Leung et al., 2006). Based on these findings, three 2MWTs are expected per month but at the expense of decreased reproducibility.

### 4.1. Clinical Implications

While the applicability of a clinician-administered walk test for monitoring purposes is limited due to the need for in-clinic assessments, wearable technology is a promising alternative enabling more frequent testing (Jehn et al., 2013; Brooks et al., 2015; Salvi et al., 2020, 2021). A few studies argued that walk testing on a monthly or even yearly basis is of limited prognostic value (Ingle et al., 2014; Prescher et al., 2016); however, the repeated testing could be beneficial when assessing the effectiveness of rehabilitation training programs or aiming at early detection of functional loss.

Out of various exercise-testing alternatives, the 6MWT is particularly attractive for implementation in wearable devices since it can be performed by most individuals except those with serious contraindications, such as recent myocardial infarction, or unstable angina (Brooks et al., 2015), or with injuries of lower extremities. Even for seriously ill patients with congestive heart failure, the self-administered 6MWT has not resulted in any falls, chest pain, shortness of breath, or need for specific medication (Brooks et al., 2015). Since unintentional walk testing does not modify casual activity habits, the proposed approach may expand the coverage of eligible populations. For example, unintentional walk testing can be a beneficial alternative for individuals with dementia, who often lose attention or have unexpected behavior during clinician-administered walk tests (Chan and Pin, 2019b).

Unintentional walk testing should not be viewed as a replacement for clinician-administered walk testing but rather as a complementary approach that allows obtaining intermediate values between walk tests performed at clinics. By combining clinician-administered and unintentional walk testing, also by including information on activity profiles (Schubert et al., 2020), the possibility opens to analyze properties of habitual and purposeful walking. Monitoring of trends in distance walked through unintentional testing might be especially useful when assessing the effectiveness and adherence to the training program at home environment, e.g., prescribed after major cardiovascular events. In case of an upward trend, it can be assumed that the patient is following a training plan and the plan is effective. In case of a downward trend, the patient might be inquired whether the training plan is performed properly, and, if necessary, is invited for clinical examination.

As already mentioned, the measurement of maximal oxygen uptake should preferably be taken to accurately assess functional capacity. Many studies show that the distance walked during the clinician-administered 6MWT is moderately linearly correlated with maximal oxygen uptake (Carter et al., 2003; Turner et al., 2004; Pulz et al., 2008; Deboeck et al., 2014). Larger correlation is observed when maximal oxygen uptake is calculated from prediction equations, complemented by easily obtainable information, such as age, sex, weight, and resting heart rate (Deka et al., 2021). While the assessment of functional capacity was out of the scope of this study, identification of a decrease in functional capacity *via* unintentional walk testing does not seem far-fetched. For instance, a study by Deboeck et al. (2014) showed that the majority of symptomatic heart failure patients with reduced functional capacity (i.e., NYHA class II and III) achieved much shorter distances during 6MWT compared to healthy subjects and asymptomatic heart failure patients (i.e., NYHA class I.). Based on this observation, unintentional walk testing can be applied to screen for those with potentially decreased functional capacity. In case the minimal distance considered of sufficient functional capacity is achieved, it is reasonable to assume that the subject will be able to walk at least the same distance during a clinician-administered test. In contrast, the subject who is unable to walk a minimal distance should be considered for clinical examination.

### 4.2. Concerns Regarding Walk Testing

Following the requirements of the guidelines for the 6MWT (American Thoracic Society, 2002), the distance walked slightly declines after the first 1–2 min and remains nearly constant during the last 4 min resulting in a marginal minute-to-minute distance variation (Motl et al., 2012; Dalgas et al., 2014). To better represent steady walking, the candidate walk tests are picked out based on the maximal cadence variation. Meanwhile, the rationale of choosing the one with the largest number of steps out of all candidates corresponds well to the goal of a clinical walk test to walk as far as possible (American Thoracic Society, 2002). Yet another possibility is to choose the walk test candidate with the lowest SD as the most representative; however, such an approach may lead to reduced reproducibility since slow steady walking can be wrongly accepted as a walk test. In addition increased variability in cadence is expected among those with movement impairments or cardiopulmonary disease. To increase the robustness to measurement errors, the 95th percentile may be preferred to the maximal value, however, such an option is only appropriate when the analysis time interval is much longer, e.g., from months to years.

Walk test performance depends on a variety of factors outside the cardiorespiratory system, namely, age, height, weight, gender, muscle strength, musculoskeletal disorders, nutritional status and cognitive function (Heresi and Dweik, 2011). Even such factors as motivation, agreeableness, anxiety, and the amount of the physical effort expended may influence the walked distance (Hearon and Harrison, 2020). No special encouragement in free-living activities may be the reason why most individuals have not reached the reference walking distance, which is in agreement with the observation in Blagev et al. (2019).

Given that an individual is not affected by boosted motivation to reach physical limits, unintentional walk testing may better reflect daily functional status. This reasoning can be supported by a well-known bias of clinician-administered walk tests known as a learning effect. Due to the learning effect, the performance tends to improve in successive tests and can result in up to 14% increase in distance walked (Chan and Pin, 2019a). This can be a major factor causing high variability in distance walked during clinician-administered walk tests (Spencer et al., 2018). Since unintentional walk testing depends on the functional status of the subject rather than on familiarity of the subject with the test, it should not be biased due to the learning effect.

The walking distance may vary widely, particularly among older individuals and those with serious health conditions. Healthy individuals over 40-years-old tend to walk distances ranging from 380 to 782 m (mean 571 m) (Casanova et al., 2011), which agrees well with our finding for this particular age group (mean 560 m). On the other hand, elderly patients with chronic heart failure achieve much shorter distances ranging from 60 to 386 m (median 232 m) (Ingle et al., 2014). In this study, patients with CVD walked markedly longer distances (mean 514 m), probably due to less serious cardiovascular conditions and superior functional status. For this reason, it was decided to limit the shortest detected distance by the minimal amount of steps, i.e., 60 per min, to ensure that ordinary walking is not accepted as a walk test; however, the minimal amount of steps per min can be set to a lower value knowing in advance the ranges of likely distances for a particular patient group.

Since an unintentional 6MWT is of an unknown walking course, the estimated distance has to be interpreted with caution as the walking path may affect the walked distance to a degree that may influence outcome prediction (Barnett et al., 2016). A study on a self-administered 6MWT, in which subjects were allowed to choose their course to walk back and forth at home environment, reported high correlation (*r* = 0.86) and acceptable distance measurement accuracy (7.6 m) compared to a clinician-administered test (Brooks et al., 2015). The accuracy of the estimated distance in the unintentional walk test remains to be established; however, study findings are in line with clinical observations since patients with CVD walked 40.4 m shorter distance on average compared with healthy subjects over 40 years. The obtained difference is larger than the reported clinically meaningful minimal difference of at least 30.5 m (Bohannon and Crouch, 2017).

### 4.3. Concerns Regarding Wrist-Worn Devices

Plenty of alternatives are available on the market, however, only a small fraction of manufacturers give access to the data, which was an important reason for choosing Fitbit wrist-worn devices. While there is a lack of studies directly comparing Fitbit Charge 2 and Alta HR, these devices likely use the same accelerometer and photoplethysmogram sensors and data processing algorithms, as they were developed by the same manufacturer. For instance, the comparison of Fitbit Charge HR and Alta HR provided nearly identical step counts during level and inclined walking (Montoye et al., 2018). In addition, no difference was observed when identifying the type and duration of physical activity (Dorn et al., 2019).

Wrist-worn devices are often prone to measurement errors observed within and across wearers (El-Amrawy and Nounou, 2015). A systematic review has shown that Fitbit devices provide an accurate step count, approximately, half the time, with a tendency to overestimate steps in free-living activities (Feehan et al., 2018). Larger errors were observed during slow walking (Wong et al., 2018), whereas decreased during fast walking, e.g., 80 m/min (Chen et al., 2016; Chow et al., 2017), which is probably related to the increased amplitude of the acceleration signal, making step detection easier. On the other hand, Fitbit devices tend to underestimate steps in a controlled environment, e.g., the absolute error of 30 steps was found in patients with Parkinson's disease who underwent 6 min walking in a lab (Lai et al., 2020). Since the proposed approach is not restricted to the step count, other modalities for distance estimation, such as GPS tracking (Salvi et al., 2020, 2021), can be employed as well.

### 4.4. Extension Opportunities

In clinical practice, the distance walked is the primary but not the only outcome measure of the 6MWT. Secondary measures may include blood oxygen saturation, heart rate, walk work (walked distance multiplied by body weight), as well as subjective experiences, such as shortness of breath and fatigue (Singh et al., 2014). While heart rate measurement is a widely used feature of commercial wrist-worn devices (Natarajan et al., 2020), the latest is also capable of estimating blood oxygen saturation (Lauterbach et al., 2020). More comprehensive measures of heart rate response, such as heart rate decrease in the 2 min recovery period after the walk test, have also been considered to supplement the assessment of the 6MWT (van Stel et al., 2001; Singh et al., 2014). Our previous work has shown that unobtrusive estimation of heart rate recovery using a wrist-worn device is in principle feasible (Sokas et al., 2019).

### 4.5. Limitations

The limitation of the study is that unintentionally performed 6MWTs were not verified by comparing to the reference clinician-administered tests. To mitigate this limitation, we compared the estimated walking distance to the reference distance calculated using the population-derived regression equation. Currently, there is no consensus on the reference 6 min walking distance despite the efforts put into finding the model which would predict the reference walking distance in the general population (Singh et al., 2014; Halliday et al., 2020). The accuracy of the majority of models is questionable due to small sample size and a single country-limited population. For this reason, we used a multiple regression equation derived from a diverse population, involving 42–76-year-old individuals from 10 centers of 7 countries (Casanova et al., 2011). We found that it was increasingly difficult for younger subjects to reach the reference walking distance in free-living activities, suggesting that this equation might be unsuitable for individuals younger than 40 years.

It should be noted that the equation for the reference walking distance includes maximal heart rate, which may alter the reference distance in particular situations. For instance, patients with the cardiopulmonary disease often have a higher heart rate during physical activity compared with healthy individuals, whereas those taking beta-blockers may have a reduced heart rate (Singh et al., 2014). In addition, Fitbit Charge 2, including its predecessor Fitbit Charge HR, may underestimate heart rate during intensive physical activity, especially above 116 bpm (Jo et al., 2016; Benedetto et al., 2018). Fortunately, the accuracy of the heart rate estimation increases during less intensive physical activity, which is common in walk tests. Based on the findings of this study, the median maximal heart rate during the 6WMT was 122 bpm (range from 68 to 189 bpm) for healthy subjects and 116 bpm (range from 82 to 174 bpm) for patients with CVD.

A stride length can be set in most wearable devices including Fitbit, unfortunately, this information was not available in this study. To compare the estimated walking distance to the reference, the number of steps was converted to the distance based on the equation involving height and age of subject. The study by Egerton et al. (2011) provides the intercept and slope coefficients for three age groups, i.e., young (18–30-year-old), mature (45–55-year-old), and older adults (>65 years old). Since the coefficients differ considerably in older adults compared with the groups of young and mature adults, we decided to use two equations to account for the influence of older age on cadence conversion to stride length. In addition, stride length depends on various other factors, and thus is highly variable even among healthy individuals (Morio et al., 2019). For example, taller individuals and men usually have a longer stride length, whereas elderly and obese individuals often have a shorter stride. As a result, they cover different distances for the same number of steps. Distances can also be underestimated during fast walking due to a longer stride length; however, this is not a crucial problem, since fast-walking individuals cover large 6MWT distances, e.g., above 550 m, which have not been related to clinical consequences (Brooks et al., 2015).

## 5. Conclusions

The study demonstrates that unintentional walk testing in free-living activities is feasible, resulting, on average, in two detected 6MWTs per month for cadence variation less than or equal to 5 steps. Based on the walked distance in the unintentional 6MWT, patients with CVD, on average, walked 46 m shorter distance compared with healthy subjects older than 40 years. Repeated monitoring of the performance in unintentional walk tests could be valuable for assessing the effectiveness of physical rehabilitation in the home environment; however, further studies are needed to validate the proposed approach by comparing it to the clinician-administered walk test.

## Data Availability Statement

The datasets presented in this article are not readily available because dataset was collected by information technology company dHealthIQ Care (Utrecht, The Netherlands). Dataset was provided to us without permission to share it publicly. Requests to access the datasets should be directed to dhealthiqcare.com.

## Ethics Statement

Ethical review and approval was not required for the study on human participants in accordance with the local legislation and institutional requirements. The patients/participants provided their written informed consent to participate in this study.

## Author Contributions

DS developed the algorithm for the detection of unintentional walk tests, contributed to study design, conducted data analysis, interpreted the results, and revised the manuscript. BP interpreted the results and revised the manuscript. AR contributed to algorithm development and revised the manuscript. VM contributed to the concept of unintentional testing using a wrist-worn device and revised the manuscript. RB contributed to the study design and revised the manuscript. AP developed the concept of unintentional walk testing, designed the study, interpreted the results, and wrote and revised the manuscript. All authors have read the submitted manuscript and approved the final version.

## Conflict of Interest

The authors declare that the research was conducted in the absence of any commercial or financial relationships that could be construed as a potential conflict of interest.

## Publisher's Note

All claims expressed in this article are solely those of the authors and do not necessarily represent those of their affiliated organizations, or those of the publisher, the editors and the reviewers. Any product that may be evaluated in this article, or claim that may be made by its manufacturer, is not guaranteed or endorsed by the publisher.
